# Hemoglobin-Conjugated Gold Nanoclusters for Qualitative Analysis of Haptoglobin Phenotypes

**DOI:** 10.3390/polym12102242

**Published:** 2020-09-29

**Authors:** Shih-Hua Tan, Sibidou Yougbaré, Hsueh-Liang Chu, Tsung-Rong Kuo, Tsai-Mu Cheng

**Affiliations:** 1 Graduate Institute of Nanomedicine and Medical Engineering, College of Biomedical Engineering, Taipei Medical University, Taipei 11031, Taiwan; b812105028@tmu.edu.tw (S.-H.T.); trkuo@tmu.edu.tw (T.-R.K.); 2 International PhD Program in Biomedical Engineering, College of Biomedical Engineering, Taipei Medical University, Taipei 11031, Taiwan; d845107003@tmu.edu.tw; 3 Institut de Recherche en Sciences de la Santé (IRSS-DRCO)/Nanoro, 03 B.P 7192 Ouagadougou 03, Burkina Faso; 4 Graduate Institute of Translational Medicine, College of Medicine and Technology, Taipei Medical University, Taipei 11031, Taiwan; szxchu@gmail.com; 5 TMU Research Center of Cancer Translational Medicine, Taipei Medical University, Taipei 11031, Taiwan

**Keywords:** haptoglobin, gold nanoclusters, hemoglobin, phenotypes, qualitative analysis

## Abstract

Designing a facile and rapid detection method for haptoglobin (Hp) phenotypes in human blood plasma is urgently needed to meet clinic requirements in hemolysis theranostics. In this work, a novel approach to qualitatively analyze Hp phenotypes was developed using a fluorescent probe of gold nanoclusters (AuNCs). Hemoglobin-conjugated (Hb)-AuNCs were successfully synthesized with blue-green fluorescence and high biocompatibility via one-pot synthesis. The fluorescence of Hb-AuNCs comes from the ligand-metal charge transfer between surface ligands of Hb and the gold cores with high oxidation states. The biocompatibility assays including cell viability and fluorescence imaging, demonstrated high biocompatibility of Hb-AuNCs. For the qualitative analysis, three Hp phenotypes in plasma, Hp 1-1, Hp 2-1, and Hp 2-2, were successfully discriminated according to changes in the fluorescence intensity and peak position of the maximum intensity of Hb-AuNCs. Our work provides a practical method with facile and rapid properties for the qualitative analysis of three Hp phenotypes in human blood plasma.

## 1. Introduction

Fluorescent nanoclusters (NCs) have been extensively utilized for biomedical applications in fields such as bioimaging, detection, and medicine [1,2,3,4,5,6]. With many kinds of metal NCs, recent advancements have focused on the development of gold NCs (AuNCs) with various surface ligand modifications for detection applications [7,8,9,10,11]. For example, glucose-conjugated AuNCs were applied as a target-specific fluorescent probe to quantitatively detect glucose metabolism rates by glucose transporters overexpressed by U-87 MG brain cancer cells [12]. Glutathione-protected AuNCs were employed to detect the biomarker of dipicolinic acid for sensing potential infections by strongly hazardous anthrax spores with a much lower limit of detection (600-fold) than the infectious dosage [13]. Hyperbranched polyethyleneimine-assisted dual-emissive AuNCs were designed to detect highly toxic cyanide anions based on changes in the fluorescence color caused by their surface Au(I) etched by cyanide anions [14]. Fluorescent AuNC-modified metal-organic frameworks have served as a highly sensitive fluorescent nanoprobe for rapid detection of nitro explosives of 2,4,6-trinitrotoluene [15]. With the conjugation of surface ligands of small molecules, polymers, and proteins, AuNCs have been intensively used for biomedical applications due to their easy surface modification, tunable fluorescence, high biocompatibility, and superior specificity [16]. Several studies have demonstrated the syntheses and applications of Hb conjugated AuNCs (Hb-AuNCs) [17,18,19,20,21,22]. However, as far as we know, there is still no design with the use of Hb-AuNCs for the qualitative detection of haptoglobin (Hp) phenotypes. The acute-phase Hp protein has three phenotypes in human blood plasma: Hp 1-1, Hp 2-1, and Hp 2-2 [23,24,25,26,27]. In the clinic, concentrations of these three Hp phenotypes are related to kidney failure, diabetes, autoimmune disorders, cardiovascular diseases, and hemolytic anemia [28,29,30,31,32,33,34]. Three ordinary approaches of spectrophotometry, immunoreactivity, and gel electrophoresis are used to measure Hp. For the spectrophotometric analysis, the concentration of Hp is determined from the change in absorption due to the formation of Hp–Hb complexes [35]. Moreover, with analysis of different absorption spectra of Hp–Hb complexes, Hp–Hb complexes, free Hb, and free Hp can be quantitatively measured with varying degrees of hemolysis. For immunoreactive assays, Hp and antisera are mixed to form precipitates, and then the precipitates are analyzed by radial immunodiffusion and nephelometry [36]. To detect the precipitates, nephelometry is a commonly utilized method by light irradiation with Hp–Hb-antibody complexes in solution to obtain reflected light for further analysis. With the use of gel electrophoresis, free Hb and Hp–Hb complexes can respectively be isolated via alkaline electrophoresis [37]. Although spectrophotometry, immunoreactivity, and gel electrophoresis have been developed to detect Hp, the qualitative analysis of the three Hp phenotypes still lacks a suitable approach. Recently, an immunoturbidimetric assay and enzyme-linked immunosorbent assay were conducted to discriminate Hp phenotypes. Designing a rapid assay for Hp phenotypes is still an urgent task to meet clinical requirements.

**Figure 1:**
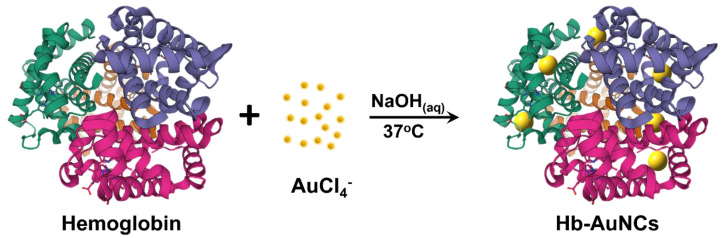
Schematic illustration of hemoglobin-conjugated gold nanoclusters (Hb-AuNCs) synthesis by a facile one-pot synthesis strategy.

In this work, for the qualitative analysis of three Hp phenotypes, Hb was chosen as a surface ligand to prepare Hb-AuNCs due to its high-affinity binding with Hp and cysteine residues. Hb-AuNCs were prepared by a facile one-pot synthesis strategy as shown in Figure 1. The structural and optical properties of Hb-AuNCs were characterized by ultraviolet–visible (UV–Vis) absorption spectroscopy, fluorescence spectroscopy, x-ray photoelectron spectroscopy (XPS), transmission electron microscopy (TEM), and Fourier transform infrared (FTIR) spectroscopy. The biocompatibility of Hb-AuNCs was assessed by a resazurin dye reduction assay and fluorescence imaging. Most importantly, fluorescent Hb-AuNCs were used to qualitatively analyze Hp phenotypes according to changes in the fluorescence spectra of Hb-AuNCs.

## 2. Materials and Methods

### 2.1. Synthesis of Hemoglobin-Conjugated Gold Nanoclusters (Hb-AuNCs)

An HAuCl_4_ aqueous solution (2.8 mM, 5 mL) (Alfa Aesar, Ward Hill, MA, USA) was added to 5 mL of an Hb aqueous solution (2 mg/mL) (Sigma-Aldrich, St. Louis, MO, USA). The reaction solution was stirred at 400 rpm in a water bath (37 °C) for 10 min. Afterward, 1 mL of an NaOH aqueous solution (1 M) (Fluka, St. Gallen, Swiss) was added to the reaction. The reaction solution containing HAuCl_4_, Hb, and NaOH was stirred at 400 rpm in a water bath (37 °C) and a dark environment for 24 h. After reacting for 24 h, the solution containing Hb-AuNCs was centrifuged at 12,000 rpm for 10 min. After centrifugation, the upper greenish-black solution containing Hb-AuNCs was collected and stored at 4 °C in the dark for subsequent experiments.

### 2.2. Characterization Techniques

UV–Vis absorption spectrometer (JASCO V-770 Spectrophotometer, Easton, MD, USA) was applied to measure the absorption of Hb-AuNCs. XPS (VG Microtech MT-500, London, United Kingdom) with Al Kα x-ray source was used to analyze the oxidation state and binding properties of Hb-AuNCs. A fluorescence spectrometer (JASCO spectrofluorometer FP-8500, Easton, MD, USA) was utilized to detect the fluorescence of Hb-AuNCs. The Systat PeakFit 4.12 (Systat Software, San Jose, CA, USA) software was employed to simulate fluorescence spectra. TEM (Hitachi HT-7700, Tokyo, Japan) was applied to characterize the morphology of the Hb-AuNCs. FTIR spectroscopy (Thermo Scientific Nicolet iS10, Waltham, MA, USA) was utilized to measure the amide groups of Hb-AuNCs.

### 2.3. Cytotoxicity Assay of Hb-AuNCs by Vero Cells

Dulbecco’s modified Eagle’s medium (DMEM) (Gibco, Waltham, MA, USA) with 10% (*v*/*v*) fetal bovine serum (FBS) (Gibco, Waltham, MA, USA) was applied to culture Vero cells. The cytotoxicity of Hb-AuNCs was measured before and after incubation with Vero cells by a resazurin dye reduction assay. Vero cells were cultured in a 96-well plate (10^4^ cells/well) for 24 h. After 24 h, the media in the 96-well plate were removed, and then a phosphate-buffered saline (PBS) solution (Thermo Fisher Scientific, Waltham, MA, USA) was utilized to wash each well cultured with Vero cells. Sequentially, Vero cells in the 96-well plates were incubated with Hb-AuNCs dispersed in DMEM. Concentrations of Hb-AuNCs of 2.7, 5.5, 11.0, 21.9, 43.8, 87.5, 175, 350, and 700 μg/mL were used to test the cytotoxicity. After 24 h of incubation, resazurin (0.02 mg/mL) (Thermo Fisher Scientific, Waltham, MA, USA) was added to each well and further incubated for an additional 4 h. The absorbances at 570 and 600 nm were measured by a plate reader (Thermo Varioskan Flash, Waltham, MA, USA). For the cell viability assay, each concentration of Hb-AuNCs was repeated independently at least eight times.

### 2.4. Measurement of Vero Cell Death by Fluorescence Imaging

To measure cell death, the SYTOX green fluorescent dye (Thermo Fisher Scientific, Waltham, MA, USA) was applied to stain dead cells for fluorescence imaging. Thus, 5 μM of SYTOX green was added to culture media of Vero cells in each well and incubated at room temperature for 15 min without light irradiation. After washing with PBS, nucleic acids of total cells were stained with Hoechst 33342 (Thermo Fisher Scientific, Waltham, MA, USA). Fluorescence images of total/dead cells were obtained with fluorescence microscopy.

### 2.5. Detection of Hp Phenotypes

The method for detecting Hp phenotypes was modified from a previous study [38]. Briefly, 7 μL of a serum sample was mixed with 5 μL Hb (8 mg/mL) and then equilibrated with 3 μL of sample buffer (0.625 M Tris-base at pH 6.8, 0.125 mg/L bromophenol blue (Sigma-Aldrich, St. Louis, MO, USA) and 50% glycerol (*v*/*v*) (BioShop Canada Inc., Burlington, ON, Canada)). The mixture was separated by non-reducing sodium dodecylsulfate polyacrylamide gel electrophoresis (SDS-PAGE) (6% acrylamide (Sigma-Aldrich, St. Louis, MO, USA) for the separating gel (pH 8.8) and 4% acrylamide for the stacking gel (pH 6.8)). After electrophoresis, the gel was stained by freshly prepared peroxidase substrate (0.05% 3,3′-diaminobenzidine (Sigma-Aldrich, St. Louis, MO, USA) and 0.07% hydrogen peroxide (Sigma-Aldrich, St. Louis, MO, USA) in PBS), and the Hp–Hb complex was visualized.

## 3. Results

### 3.1. Optical and Structural Characterizations of Hb-AuNCs

**Figure 2:**
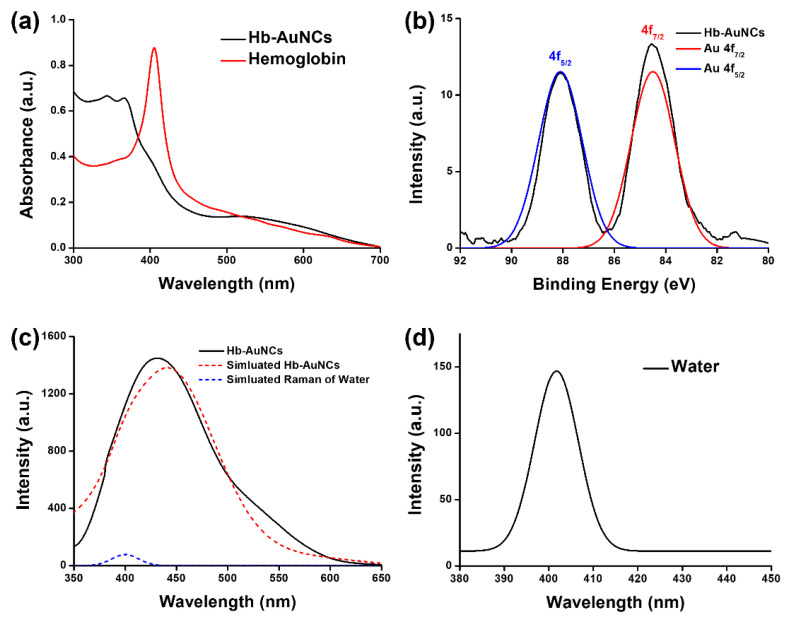
(**a**) Ultraviolet–visible (UV–Vis) absorption spectra of hemoglobin and hemoglobin-conjugated gold nanocomposites (Hb-AuNCs); (**b**) X-ray photoelectron (XPS) spectra of Hb-AuNCs (black line), the simulated peak of Au 4f_5/2_ (blue line) and the simulated peak of Au 4f_7/2_ (red line); (**c**) fluorescence spectrum of Hb-AuNCs composed of the simulated fluorescence spectrum of Hb-AuNCs and the simulated Raman signal of water. The excitation wavelength was 314 nm; (**d**) Raman signal of water was measured with the excitation wavelength of 314 nm by fluorescence spectrometer.

In Figure 2a, the UV–Vis absorption spectrum of Hb exhibited a strong Soret band at a wavelength of 405 nm. After the formation of Hb-AuNCs, the signal intensity of the Soret band decreased, and its peak position also revealed a blue-shift from 405 to 367 nm, as shown in the UV–Vis absorption spectrum of Hb-AuNCs in Figure 2a. The decrease in the signal intensity and blue-shift of the peak position can be attributed to a change in the hydrophobicity of heme groups due to the formation of Hb-AuNCs [39]. Furthermore, there was no obvious surface plasmon absorption peak at a wavelength of ~520 nm due to the absence of gold nanoparticles [40]. The disappearance of the surface plasmon absorption of gold nanoparticles can be ascribed to Hb-AuNCs exhibiting high oxidation states of gold cores, which produced a shortage of free electrons to generate coherent oscillations [41,42,43]. To further examine the oxidation state and binding properties, XPS spectrum of Hb-AuNCs were analyzed. In the XPS spectrum of Hb-AuNCs in Figure 2b, the peaks of Au 4f_5/2_ and Au 4f_7/2_ were separately located at 88.1 and 84.5 eV. For bulk gold, peaks of Au 4f_5/2_ and Au 4f_7/2_ were located at 87.4 and 84.0 eV, respectively. Compared to bulk gold, the positive shifts of Au 4f_5/2_ and Au 4f_7/2_ indicated that Hb-AuNCs contained a lot of gold with high oxidation states in their cores, corresponding to the results of the UV–Vis absorption spectrum of Hb-AuNCs [44]. Moreover, the fluorescence spectrum of Hb-AuNCs was measured. Under an excitation wavelength of 314 nm, Hb-AuNCs exhibited a maximum intensity of fluorescence at a wavelength of 430 nm as shown in Figure 2c. A small shoulder was observed at a wavelength of 408 nm due to the Raman signal of water. Raman signal of water was measured with the excitation wavelength of 314 nm by fluorescence spectrometer, as shown in Figure 2d. Based on the peak simulations, the fluorescence spectrum of Hb-AuNCs was separated into two peaks including a fluorescence spectrum of Hb-AuNCs and the Raman signal of water, as shown in Figure 2c. The fluorescence of AuNCs was proven to be due to the ligand-metal charge transfer between thiolate ligands and the core of AuNCs [45]. For Hb-AuNCs, based on the ligand-metal charge transfer, electrons in the ligand of Hb were delivered to the gold cores with high oxidation states to generate fluorescence.

**Figure 3:**
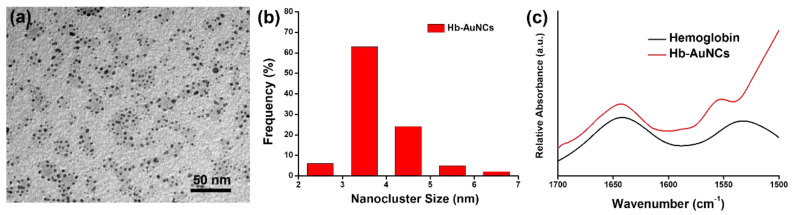
(**a**) Transmission electron microscopy (TEM) image of hemoglobin-conjugated gold nanoclusters (Hb-AuNCs); (**b**) histogram of the size distribution of Hb-AuNCs; (**c**) Fourier transform infrared (FTIR) spectra of Hb and Hb-AuNCs.

In the TEM image of Figure 3a, Hb-AuNCs revealed an approximately globular shape. As shown in the histogram of Figure 3b, the size distribution of Hb-AuNCs was systematically counted according to 100 nanoclusters in the TEM image. The average size of Hb-AuNCs was calculated to be 3.7 nm. Overall, from a combination of optical and structural characterizations, we determined that fluorescent Hb-AuNCs were successfully prepared by a facile hydrothermal approach. FTIR spectra of Hb and Hb-AuNCs were applied to investigate their characteristic amide I and amide II bands. The amide I band (1600–1700 cm^−1^) can be ascribed to the stretching vibrations of CO from peptide linkages in the protein backbone. The amide II band (1500–1600 cm^−1^) was associated with NH bending and CN stretching vibrations. As shown in the FTIR spectra of Figure 3c, Hb exhibited characteristic IR bands of amide I and amide II located at 1642 and 1534 cm^−1^, respectively. After formation of Hb-AuNCs, the amide I band showed no significant change. However, the amide II band revealed the increase of intensity and the peak position was shifted from 1534 cm^−1^ to 1553 cm^−1^. Previous studies have demonstrated that the increase in peak intensity and the shift of peak position can be attributed to the formation of Hb-AuNCs. Based on the results of the FTIR spectra, the conjugation between Hb and AuNCs was successfully obtained [46,47].

### 3.2. Biocompatibility Assays of Hb-AuNCs

**Figure 4:**
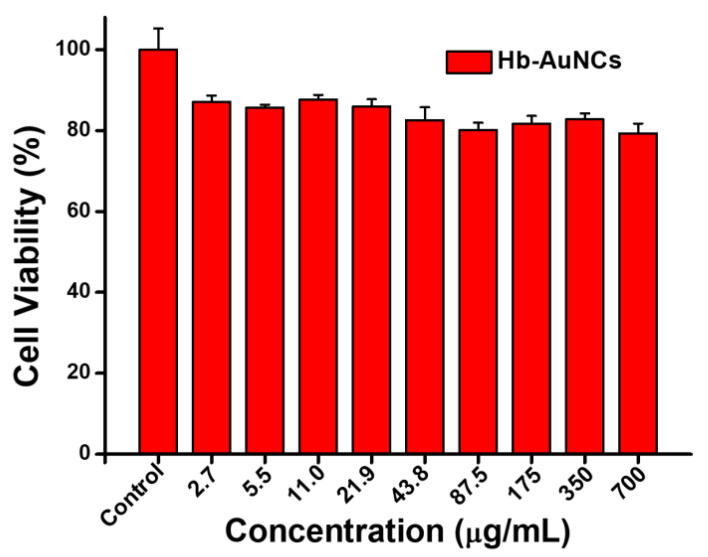
Cell viabilities of hemoglobin-conjugated gold nanoclusters (Hb-AuNCs) at concentrations of 2.7~700 μg/mL via a resazurin dye reduction assay.

For further application as a biomedical sensor, the cell viability of Hb-AuNCs was evaluated by a resazurin dye reduction assay. Vero cells cultured in DMEM without Hb-AuNCs were used as the control. Water-soluble Hb-AuNCs at different concentrations of 2.7, 5.5, 11.0, 21.9, 43.8, 87.5, 175, 350, and 700 μg/mL were separately incubated with Vero cells to examine cell viability. As shown in Figure 4, all cell viabilities of Hb-AuNCs at concentrations of 2.7~700 μg/mL were >80%. High cell viabilities indicated that Hb-AuNCs exhibited no significant cytotoxicity.

**Figure 5:**
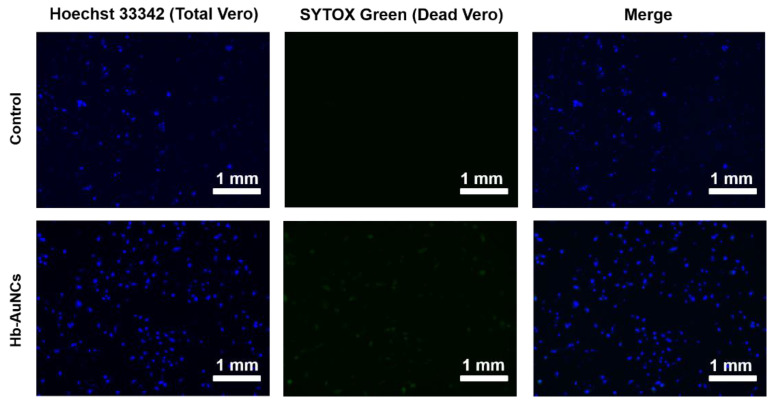
Fluorescence images of Vero cells incubated with hemoglobin-conjugated gold nanoclusters (Hb-AuNCs). In the control experiments, Vero cells were cultured with Dulbecco’s modified Eagle’s medium (DMEM).

To further investigate the cytotoxicity of Hb-AuNCs, fluorescence images of Vero cells incubated with Hb-AuNCs were examined. In Figure 5, blue and green pseudocolors indicate fluorescence signals of total Vero cells including both live and dead cells (stained with Hoechst 33342) and dead Vero cells (stained with SYTOX green), respectively. For the control, not incubated with Hb-AuNCs, Vero cells homogeneously grew as indicated by the blue pseudocolor as shown in Figure 5 (top left). There was no detectable green pseudocolor, indicating no dead Vero cells in the control, as shown in Figure 5 (top middle). After incubation with Hb-AuNCs, Vero cells also revealed homogeneous growth as indicated by the blue pseudocolor, as shown in Figure 5 (bottom left). There were only slight signals of green pseudocolor in the Vero cells incubated with Hb-AuNCs, indicating high biocompatibility of Hb-AuNCs, as shown in Figure 5 (bottom middle). Overall, the results of the cell viability assays suggest that Hb-AuNCs could be a potential fluorescent probe for biomedical sensors based on their biocompatibility.

### 3.3. Qualitative Analysis of Hp Phenotypes by Hb-AuNCs

**Figure 6:**
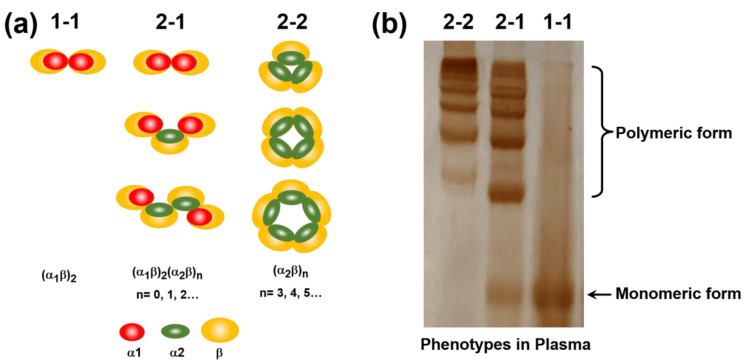
(**a**) Schematic illustration of haptoglobin (Hp) phenotypes of Hp 1-1, Hp 2-1, and Hp 2-2; (**b**) native-PAGE of plasma phenotypes of Hp 1-1, Hp 2-1, and Hp 2-2.

There are three different phenotypes of haptoglobin of Hp 1-1, Hp 2-1, and Hp 2-2, as shown in Figure 6a. Hp 1-1 is composed of two units of α1β. The β chain is formed of 245 amino-acid residues (40 kDa), and the α1 chain is formed of 83 amino-acid residues (9 kDa) [38]. In Hp 2-1, the α1β chain is conjugated with different units of α2β and finally conjugated with α1β to form (α1β)_2_(α2β)_n_ (*n* = 0, 1, 2…). Hp 2-2 is composed of various numbers of (α2β)_n_ (*n* = 3,4,5…) to form a linear structure, and the linear structure sequentially forms the cyclic structure of Hp 2-2. For the native-PAGE of Figure 6b, plasma revealed phenotypes including the monomeric form of Hp 1-1 and polymeric patterns of Hp 2-1 and Hp 2-2. The monomeric form of Hp 1-1 was composed of (α1β)_2_ as indicated in Figure 6a. For Hp 2-1, the monomeric form of (α1β)_2_ and polymeric form of (α1β)_2_(α2β)_1_ were observably revealed in the native-PAGE results in Figure 6b. Moreover, Hp 2-2 also showed polymeric forms of (α2β)_n_. For subsequent phenotype detection, three different phenotypes of Hp 1-1, Hp 2-1, and Hp 2-2 in plasma were respectively measured by Hb-AuNCs.

**Figure 7:**
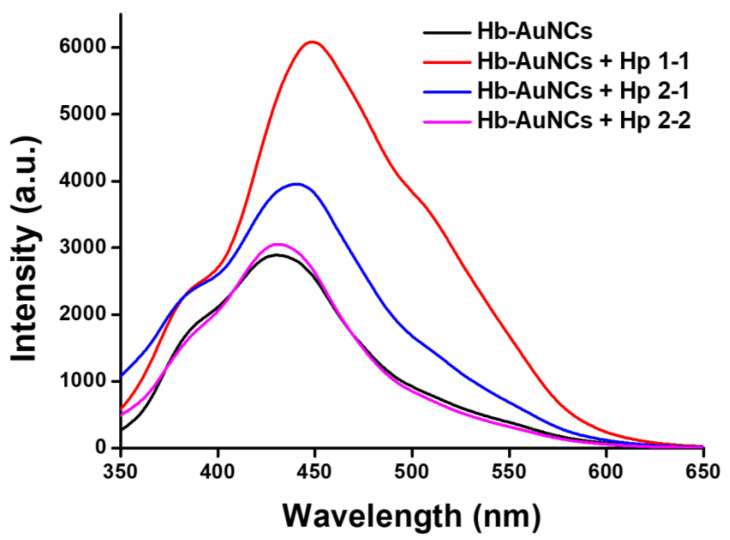
Fluorescence spectra of hemoglobin-conjugated gold nanoclusters (Hb-AuNCs) before and after adding Hp 1-1, Hp 2-2, and Hp 2-2.

To detect Hp phenotypes, human blood plasma with three different Hp phenotypes of Hp 1-1, Hp 2-1, and Hp 2-2 was prepared. In this work, plasma (5 μL) with three different Hp phenotypes was respectively added to 100 μL of an Hb-AuNC solution (700 μg/mL), and then the mixtures were mixed by vortexing for 5 min. After vortexing for 5 min, the fluorescence spectra of the mixtures containing Hb-AuNCs and plasma were measured, as shown in Figure 7. Compared to the fluorescence spectrum of Hb-AuNCs, the fluorescence spectrum of Hb-AuNCs with Hp 1-1 revealed an increase in the maximum intensity from 2892 to 6082, and the peak position of the maximum intensity was red shifted from 430 to 448 nm. For Hp 2-1 with Hb-AuNCs, the fluorescence spectrum showed an increase in the maximum intensity from 2892 to 3953, and the peak position of the maximum intensity was red shifted from 430 to 439 nm. After Hb-AuNCs were mixed with Hp 2-2, the fluorescence spectrum exhibited a slight increase in the maximum intensity from 2892 to 3054, and the peak position of the maximum intensity was red shifted from 430 to 431 nm. A previous study demonstrated that the domain of Hp serine protease exhibited extensive conjugation with the α- and β-subunits of Hb to form tightly bound Hp–Hb complexes [48]. Furthermore, cysteine residues of proteins are an important ligand to encapsulate and stabilize AuNCs due to the strong Au–S bonds between the surface of AuNCs and thiol groups of cysteine. The number of cysteines can cause a shift in the fluorescence spectrum of AuNCs [49]. Moreover, concentrations of Hp 1-1, Hp 2-1, and Hp 2-2 in plasma were demonstrated to be in the order of Hp 1-1 (184 ± 42 mg/dL) > Hp 2-1 (153 ± 55 mg/dL) > Hp 2-2 (93 ± 54 mg/dL) [38]. Therefore, after Hb-AuNCs were mixed with Hp, Hp-Hb-AuNCs were formed. With the increase in cysteines, Hp-Hb-AuNCs revealed an increase in the maximum fluorescence intensity and a red shift of the peak position of the maximum intensity compared to that of Hb-AuNCs. With the highest concentration in plasma, Hp 1-1 conjugated with Hb-AuNCs exhibited the strongest fluorescence intensity and greatest red shift of the peak position of the maximum intensity compared to those of Hp 2-1 and Hp 2-2 conjugated with Hb-AuNCs. Overall, based on changes in the fluorescence intensity and the peak position of the maximum intensity of Hp-Hb-AuNCs, the three different Hp phenotypes in plasma of Hp 1-1, Hp 2-1, and Hp 2-2 were successfully qualitatively analyzed by fluorescent Hb-AuNCs.

## 4. Conclusions

In summary, fluorescent Hb-AuNCs were successfully synthesized via a facile hydrothermal synthesis method. With the formation of Hb-AuNCs, the absorption spectrum of Hb-AuNCs revealed a decrease in the signal intensity and a blue-shift from 405 to 367 nm of the Hb Soret band due to a change in the hydrophobicity of the heme groups. Based on results of the XPS analysis, Hb-AuNCs exhibited peaks of Au 4f_5/2_ and Au 4f_7/2_ separately located at 88.1 and 84.5 eV because of a lot of gold with high oxidation states in their cores. The fluorescence spectrum of Hb-AuNCs demonstrated the maximum fluorescence intensity at a wavelength of 430 nm under an excitation wavelength of 314 nm. For structural characterization, Hb-AuNCs showed an approximately globular shape, and their average size was calculated to be 3.7 nm. Moreover, Hb-AuNCs were verified to have high biocompatibility according to a cell viability assay and fluorescence images of Vero cells. To detect the three different Hp phenotypes, fluorescent Hb-AuNCs were successfully qualitatively analyzed in plasma because of the formation of Hp-Hb-AuNCs. Overall, our work demonstrated that fluorescent Hb-AuNCs can be applied for the clinical diagnosis of three different Hp phenotypes.

## References

[1] Zhang H., Liu H., Tian Z., Lu D., Yu Y., Cestellos-Blanco S., Sakimoto K.K., Yang P. (2018). Bacteria photosensitized by intracellular gold nanoclusters for solar fuel production. Nat. Nanotechnol..

[2] Sakimoto K.K., Zhang S.J., Yang P. (2016). Cysteine–cystine photoregeneration for oxygenic photosynthesis of acetic acid from CO_2_ by a tandem inorganic–biological hybrid system. Nano Lett..

[3] Weng B., Lu K.-Q., Tang Z., Chen H.M., Xu Y.-J. (2018). Stabilizing ultrasmall Au clusters for enhanced photoredox catalysis. Nat. Commun..

[4] Wen F., Dong Y., Feng L., Wang S., Zhang S., Zhang X. (2011). Horseradish peroxidase functionalized fluorescent gold nanoclusters for hydrogen peroxide sensing. Anal. Chem..

[5] Liu C.-L., Wu H.-T., Hsiao Y.-H., Lai C.-W., Shih C.-W., Peng Y.-K., Tang K.-C., Chang H.-W., Chien Y.-C., Hsiao J.-K. (2011). Insulin-directed synthesis of fluorescent gold nanoclusters: Preservation of insulin bioactivity and versatility in cell imaging. Angew. Chem. Int. Ed..

[6] Wu X., He X., Wang K., Xie C., Zhou B., Qing Z. (2010). Ultrasmall near-infrared gold nanoclusters for tumor fluorescence imaging in vivo. Nanoscale.

[7] Li C.-H., Kuo T.-R., Su H.-J., Lai W.-Y., Yang P.-C., Chen J.-S., Wang D.-Y., Wu Y.-C., Chen C.-C. (2015). Fluorescence-guided probes of aptamer-targeted gold nanoparticles with computed tomography imaging accesses for in vivo tumor resection. Sci. Rep..

[8] Chen L.Y., Wang C.W., Yuan Z., Chang H.T. (2015). Fluorescent gold nanoclusters: Recent advances in sensing and imaging. Anal. Chem..

[9] Lakkakula J.R., Divakaran D., Thakur M., Kumawat M.K., Srivastava R. (2018). Cyclodextrin-stabilized gold nanoclusters for bioimaging and selective label-free intracellular sensing of Co^2+^ ions. Sens. Actuators B Chem..

[10] Xie Y., Xianyu Y., Wang N., Yan Z., Liu Y., Zhu K., Hatzakis N.S., Jiang X. (2018). Functionalized gold nanoclusters identify highly reactive oxygen species in living organisms. Adv. Funct. Mater..

[11] Zang J., Li C., Zhou K., Dong H., Chen B., Wang F., Zhao G. (2016). Nanomolar Hg^2+^ detection using β-lactoglobulin-stabilized fluorescent gold nanoclusters in beverage and biological media. Anal. Chem..

[12] Cheng T.M., Chu H.L., Lee Y.C., Wang D.Y., Chang C.C., Chung K.L., Yen H.C., Hsiao C.W., Pan X.Y., Kuo T.R. (2018). Quantitative analysis of glucose metabolic cleavage in glucose transporters overexpressed cancer cells by target-specific fluorescent gold nanoclusters. Anal. Chem..

[13] Halawa M.I., Li B.S., Xu G.B. (2020). Novel synthesis of thiolated gold nanoclusters induced by lanthanides for ultrasensitive and luminescent detection of the potential anthrax spores’ biomarker. ACS Appl. Mater. Interfaces.

[14] Yang H.W., Yang Y., Liu S.L., Zhan X.X., Zhou H., Li X.S., Yuan Z.Q. (2020). Ratiometric and sensitive cyanide sensing using dual-emissive gold nanoclusters. Anal. Bioanal. Chem..

[15] Zhao Y., Pan M., Liu F., Liu Y.H., Dong P., Feng J., Shi T.H., Liu X.Q. (2020). Highly selective and sensitive detection of trinitrotoluene by framework-enhanced fluorescence of gold nanoclusters. Anal. Chim. Acta.

[16] Ungor D., Dékány I., Csapó E. (2019). Reduction of tetrachloroaurate (iii) ions with bioligands: Role of the thiol and amine functional groups on the structure and optical features of gold nanohybrid systems. Nanomaterials.

[17] Sarparast M., Molaabasi F., Ghazfar R., Ashtiani M.M., Qarai M.B., Taherpour A.A., Amyab S.P., Shamsipur M. (2019). Efficient ethanol oxidation by hemoglobin-capped gold nanoclusters: The critical role of fe in the heme group as an oxophilic metal active site. Electrochem. Commun..

[18] Shamsipur M., Samandari L., Farzin L., Sarparast M., Molaabasi F., Mousazadeh M.H. (2020). Dual-modal label-free genosensor based on hemoglobin@ gold nanocluster stabilized graphene nanosheets for the electrochemical detection of BCR/ABL fusion gene. Talanta.

[19] Li Y., Peng W., You X. (2017). Determination of dopamine by exploiting the catalytic effect of hemoglobin–stabilized gold nanoclusters on the luminol–naio 4 chemiluminescence system. Microchim. Acta.

[20] Shamsipur M., Molaabasi F., Shanehsaz M., Moosavi-Movahedi A.A. (2015). Novel blue-emitting gold nanoclusters confined in human hemoglobin, and their use as fluorescent probes for copper (ii) and histidine. Microchim. Acta.

[21] Molaabasi F., Hosseinkhani S., Moosavi-Movahedi A.A., Shamsipur M. (2015). Hydrogen peroxide sensitive hemoglobin-capped gold nanoclusters as a fluorescence enhancing sensor for the label-free detection of glucose. RSC Adv..

[22] Shamsipur M., Pashabadi A., Molaabasi F. (2015). A novel electrochemical hydrogen peroxide biosensor based on hemoglobin capped gold nanoclusters–chitosan composite. RSC Adv..

[23] Hochberg I., Roguin A., Nikolsky E., Chanderashekhar P.V., Cohen S., Levy A.P. (2002). Haptoglobin phenotype and coronary artery collaterals in diabetic patients. Atherosclerosis.

[24] Bamm V.V., Tsemakhovich V.A., Shaklai M., Shaklai N. (2004). Haptoglobin phenotypes differ in their ability to inhibit heme transfer from hemoglobin to LDL. Biochemistry.

[25] Langlois M.R., Martin M.E., Boelaert J.R., Beaumont C., Taes Y.E., De Buyzere M.L., Bernard D.R., Neels H.M., Delanghe J.R. (2000). The haptoglobin 2-2 phenotype affects serum markers of iron status in healthy males. Clin. Chem..

[26] Levy A.P., Hochberg I., Jablonski K., Resnick H.E., Lee E.T., Best L., Howard B.V. (2002). Haptoglobin phenotype is an independent risk factor for cardiovascular disease in individuals with diabetes: The strong heart study. J. Am. Coll. Cardiol..

[27] Levy A.P., Roguin A., Hochberg I., Herer P., Marsh S., Nakhoul F.M., Skorecki K. (2000). Haptoglobin phenotype and vascular complications in patients with diabetes. N. Engl. J. Med..

[28] Maeda N., Smithies O. (1986). The evolution of multigene families-human haptoglobin genes. Annu. Rev. Genet..

[29] Gutteridge J.M.C. (1987). The antioxidant activity of haptoglobin towards hemoglobin-stimulated lipid-peroxidation. Biochim. Biophys. Acta.

[30] Melamed-Frank M., Lache O., Enav B.I., Szafranek T., Levy N.S., Ricklis R.M., Levy A.P. (2001). Structure-function analysis of the antioxidant properties of haptoglobin. Blood.

[31] Ebersole J.L., Machen R.L., Steffen M.J., Willmann D.E. (1997). Systemic acute-phase reactants, c-reactive protein and haptoglobin, in adult periodontitis. Clin. Exp. Immunol..

[32] Langlois M.R., Delanghe J.R. (1996). Biological and clinical significance of haptoglobin polymorphism in humans. Clin. Chem..

[33] Dobryszycka W. (1997). Biological functions of haptoglobin-new pieces to an old puzzle. Eur. J. Clin. Chem. Clin. Biochem..

[34] Okuyama N., Ide Y., Nakano M., Nakagawa T., Yamanaka K., Moriwaki K., Murata K., Ohigashi H., Yokoyama S., Eguchi H. (2006). Fucosylated haptoglobin is a novel marker for pancreatic cancer: A detailed analysis of the oligosaccharide structure and a possible mechanism for fucosylation. Int. J. Cancer.

[35] Bong-Sop S., Dae-Myung J. (1984). Simple spectrophotometric determination of haptoglobin level in serum. Clin. Chim. Acta.

[36] Braun H., Aly F. (1969). Problems in the quantitative estimation of human serum haptoglobin by single radial immunodiffusion. Clin. Chim. Acta.

[37] Van Lente F., Marchand A., Galen R.S. (1979). Evaluation of a nephelometric assay for haptoglobin and its clinical usefulness. Clin. Chem..

[38] Cheng T.-M., Pan J.-P., Lai S.-T., Kao L.-P., Lin H.-H., Mao S.J. (2007). Immunochemical property of human haptoglobin phenotypes: Determination of plasma haptoglobin using type-matched standards. Clin. Biochem..

[39] Dou X., Yuan X., Yu Y., Luo Z., Yao Q., Leong D.T., Xie J. (2014). Lighting up thiolated Au@ Ag nanoclusters via aggregation-induced emission. Nanoscale.

[40] Shamsipur M., Molaabasi F., Sarparast M., Roshani E., Vaezi Z., Alipour M., Molaei K., Naderi-Manesh H., Hosseinkhani S. (2018). Photoluminescence mechanisms of dual-emission fluorescent silver nanoclusters fabricated by human hemoglobin template: From oxidation-and aggregation-induced emission enhancement to targeted drug delivery and cell imaging. ACS Sustain. Chem. Eng..

[41] Shang L., Dong S., Nienhaus G.U. (2011). Ultra-small fluorescent metal nanoclusters: Synthesis and biological applications. Nano Today.

[42] Muhammed M.A.H., Verma P.K., Pal S.K., Kumar R.A., Paul S., Omkumar R.V., Pradeep T. (2009). Bright, NIR-emitting Au23 from Au25: Characterization and applications including biolabeling. Chem. Eur. J..

[43] Wu Z., Jin R. (2010). On the ligand’s role in the fluorescence of gold nanoclusters. Nano Lett..

[44] Negishi Y., Nobusada K., Tsukuda T. (2005). Glutathione-protected gold clusters revisited: Bridging the gap between gold (i)−thiolate complexes and thiolate-protected gold nanocrystals. J. Am. Chem. Soc..

[45] Luo Z., Yuan X., Yu Y., Zhang Q., Leong D.T., Lee J.Y., Xie J. (2012). From aggregation-induced emission of Au (i)–thiolate complexes to ultrabright Au (0)@ Au (i)–thiolate core–shell nanoclusters. J. Am. Chem. Soc..

[46] Ungor D., Horváth K., Dékány I., Csapó E. (2019). Red-emitting gold nanoclusters for rapid fluorescence sensing of tryptophan metabolites. Sens. Actuators B Chem..

[47] Del Caño R., Mateus L., Sánchez-Obrero G., Sevilla J.M., Madueño R., Blázquez M., Pineda T. (2017). Hemoglobin bioconjugates with surface-protected gold nanoparticles in aqueous media: The stability depends on solution pH and protein properties. J. Colloid Interface Sci..

[48] Andersen C.B.F., Torvund-Jensen M., Nielsen M.J., de Oliveira C.L.P., Hersleth H.-P., Andersen N.H., Pedersen J.S., Andersen G.R., Moestrup S.K. (2012). Structure of the haptoglobin–haemoglobin complex. Nature.

[49] Xu Y., Sherwood J., Qin Y., Crowley D., Bonizzoni M., Bao Y. (2014). The role of protein characteristics in the formation and fluorescence of au nanoclusters. Nanoscale.

